# The Board of Censors and Medical Education

**Published:** 1887-07

**Authors:** 


### Editorial.

### *THE BOARD OF CENSORS AND MEDICAL EDUCATION.*

The semi-annual meeting of the Erie County Medical Society, held in this city June 13th, was a notable one. Rumors had been in circulation that the Censors intended to present a report which would afford an interesting subject for discussion. The capacity of this committee for aggressive work, whether such work was just or otherwise, has been tried in the previous history of the society. At this meeting, the Censors entered upon a new field of official inquiry, and branched off into a long and justly-severe criticism of the ignorance prevailing among medical men, and the necessity of a good "English academical education, and a knowledge of Latin," for all applicants who aim to enter upon the study of medicine.

The recent examination held by the Civil Service Commission for the position of District Health Physician, opened the way for an exposure of the facts alluded to in this report. So great was their astonishment at the result of the aforesaid examination, that the Censors "deemed it their duty to take official action thereon, and report the same to the society." They further report that "they had gained reliable information that all the applicants" who made such a lamentable display of ignorance "were graduates of *recognized* medical colleges." They confess that nearly all of said applicants "have so signally failed in the attempt to pass the examination, and have shown such gross ignorance of medical science, and a *total* want of literary and classical education, their written work having been so poor and defective in spelling that on the first examination not one of them reached the lowest standard of sixty, set down by the Commissioners, that *all* were rejected." The Board of Health, in its mercy for the poor ignoramuses, requested the Examiners not to publish the names of the rejected applicants, fearing such publication might injure them in the estimation of the public.

In proof of this severe judgment, the report includes a letter written by one of these illiterate applicants, the grammar and orthography of which fully sustain the severe judgment they render.

The Censors further report that, "taking these deplorable facts into consideration, your Board have come to the conclusion that the by-laws of the Medical Society of Erie County have been disregarded and flagrantly violated by some of its members, in having admitted to their offices, as students of medicine such applicants without the necessary certificates of the Board of Censors as prescribed for in our by-laws, or, on the other hand, the State Law, the charter of the medical college has been violated through its faculty granting a diploma to a candidate who has not produced a certificate of his preceptor, setting forth that he has studied medicine with him in compliance with the requirements embodied in the charter of every medical college in this State."

Finally, the Censors favor the bill, defeated a few years ago, establishing a State Board of Medical Examiners, as the only measure to correct the abuses heaped upon the public by certain diploma mills. Our readers will recall the favorable attitude of the JOURNAL towards this measure. We quite agree with the Censors in the opinion that the passage of this bill was defeated by some persons "actuated by mercenary motives and self-interest."

The report, from which we have quoted so largely, emphasizes three important facts, which have been long recognized by the the profession, viz.: (1) The illiteracy of many graduates in medicine; (2) the commercial spirit controlling medical colleges; (3) the necessity of a State Board of Medical Examiners. This confession constitutes an interesting chapter in the medical politics of this city. We recall some bitter experiences within the past few years connected with the County Society, and we are led to wonder as to the causes of the conversion of the Members of the Board of Censors to sound ethical and professional principles. The recognition of the facts forcibly brought out in this report actuated an influential body of medical men in the organization of the medical department of Niagara University, devoted to higher medical education. The flagrant abuses prevailing in certain schools were as great then as now. Indeed, some of the men, whose ignorance was displayed in the recent examination to a degree which made the cheeks of the Censors blush with shame, had unfurled the insignia of their profession to the breeze, under the seal of chartered medical schools, managed upon the principle of more graduates, more shekels to the common treasury. The sensibilities of these gentlemen were singularly obtuse to questions of this character at that time, in view of the urgent necessity then existing to stamp out the organization of a rival college, which had been called into existence to rescue the profession from the disgrace of the very abuses of which they now complain.

In conclusion, we beg to bring out the facts which called forth the report upon which we have commented. The graduates whose ignorance amazed the Censors, were nearly all alumni of the Buffalo Medical College. We make two honorable exceptions, in the persons of two highly-educated medical men, who received their diplomas from Homœopathic colleges, both of whom passed the highest in the examinations; one received the coveted appointment, and the other failed only for the reason that he had not resided in the district the time required by law.

What a ridiculous position, in its relations to medical education, the Censors have placed the Buffalo Medical College, of which they are nearly all graduates, and for many years earnest defenders! Compare the results of this test of the work done for the profession, as brought out by the examination, with the work of Schools of Medicine with which they are not in affiliation, and which many affect to despise, and the comparison forms a judgment of an institution which, after nearly a half century of labor, is justly criticised in the house of its own friends.

In this connection, it may be said that, numerically, the alumni of Buffalo Medical College are in the ascendency in the County Society. Many of them have gained an honored position in the profession for their attainments and skill. It must be humiliating for men of such high character and conceded professional ability to recognize as fellow alumni individuals plainly illiterate, and unworthy of the degree of Doctor of Medicine! Let us hope that this emphatic protest from a Society composed largely of its alumni, who have the interests of the profession and the college equally at heart, will awaken this corporation to a realizing sense of its duty to the profession and the public in the future administration of the trust reposed in it.

